# The experience and impact of patient’s suicide on psychiatrists in Malaysia

**DOI:** 10.1371/journal.pone.0356052

**Published:** 2026-08-13

**Authors:** Muhammad Hanif Abd Latif, Nur Iwana Abdul Taib, Abdul Hakem Zahari, Tuti Iryani Mohd Daud, Ravivarma Rao Panirselvam, Johari Khamis, Ahmad Rostam Md Zin, Hazli Zakaria

**Affiliations:** 1 Department of Psychiatry, Faculty of Medicine, Universiti Kebangsaan Malaysia, Kuala Lumpur, Malaysia; 2 Department of Psychiatry, Hospital Canselor Tuanku Muhriz, Universiti Kebangsaan Malaysia, Kuala Lumpur, Malaysia; 3 Department of Psychological Medicine, Faculty of Medicine and Health Sciences, Universiti Malaysia Sarawak, Kota Samarahan, Sarawak, Malaysia; 4 Department of Psychiatry and Mental Health, Hospital Selayang, Selangor, Malaysia; 5 Department of Psychiatry and Mental Health, Hospital Miri, Sarawak, Malaysia; 6 Forensic Psychiatry Division, Hospital Permai, Johor Bahru, Johor, Malaysia; 7 Department of Psychiatry and Mental Health, Hospital Sultanah Bahiyah, Alor Setar, Kedah, Malaysia; 8 Alaminda Clinic, Shah Alam, Selangor, Malaysia; University of Padova: Universita degli Studi di Padova, ITALY

## Abstract

**Introduction:**

Patient suicide is one of the most distressing clinical events a psychiatrist can encounter, yet research in lower-middle-income countries remains limited. The main objectives of this study were to examine the emotional and professional impact of patient suicide on psychiatrists in Malaysia and to identify their support needs following such events.

**Methods:**

A cross-sectional survey was conducted among psychiatrists working in government, university, and private healthcare settings across Malaysia. Participants completed an online questionnaire exploring experiences of patient suicide, emotional responses, effects on clinical practice, and perceptions of the availability and usefulness of support after the incidents.

**Results:**

A total of 120 psychiatrists participated, of whom 79% had experienced at least one patient who died by suicide, with 53.7% reporting that the most impactful event occurred within the first five years of practice. Most respondents reported moderate to high emotional impact, with a median rating of 7 out of 10. Over half of psychiatrists (56.8%) experienced a detrimental effect on clinical confidence, while 55.8% reported increased vigilance in suicide risk assessment and documentation. Female psychiatrists reported significantly higher perceived responsibility for the patient’s death than male psychiatrists at the time of the event (p = 0.01). Despite substantial distress, 95.7% of psychiatrists did not take any time off work, and only 16.8% accessed external support. More than half (53.7%) reported being unfamiliar with procedures following a patient suicide. Supportive leadership, peer support, and clear organisational processes were perceived as beneficial, whereas blame and a lack of structured support were regarded as unhelpful.

**Conclusions:**

Patient suicide is a common and emotionally challenging experience for psychiatrists in Malaysia, especially during the early stages of their careers. The findings reveal gaps in postvention support and highlight the need for structured, proactive frameworks to support clinician wellbeing and function after a patient’s suicide.

## 1. Introduction

Suicide constitutes a significant public health concern worldwide, including Malaysia [1,2]. While the profound impact of suicide on families and communities has been broadly acknowledged, greater focus has been directed toward its repercussions on healthcare professionals [3]. For psychiatrists specifically, patient suicide is considered one of the most distressing adverse clinical events encountered in professional practice. It has the potential to influence emotional well-being, professional identity, and subsequent clinical decision-making [4].

International studies indicate that between 46% and 78% of psychiatrists have encountered the experience of losing a patient to suicide during their professional careers [4,5]. Exposure to patient suicide has been linked to a spectrum of psychological responses, including grief, guilt, shame, self-doubt, and symptoms akin to post-traumatic stress [6,7]. In line with previous research, the emotional impact of a patient’s suicide appears to be more intense when it occurs during psychiatric training than after graduation, with trainees and early-career psychiatrists reported to be particularly vulnerable [8–10]. The patient’s suicide, which happened within the first 10 years of practice during residency, was reported as being extremely distressing [11].

In this regard, the experience of a psychiatrist transcends mere professional setback, often reflecting the bereavement processes observed in other survivors of suicide loss [5,12]. According to the conceptual framework proposed by Cerel et al., individuals affected by suicide can be classified according to their level of attachment and the personal impact of the loss, ranging from those merely exposed to suicide to those considered bereaved by it [13]. Comprehending the position of psychiatrists along this continuum is crucial, as the intensity of the therapeutic relationship frequently influences the severity of subsequent emotional and professional trauma.

Beyond personal grief, it can significantly impact clinicians’ confidence, professional identity, and emotional well-being. While the primary tragedy concerns the patient and their loved ones, the effect on the treating clinician is substantial, commonly leading to a condition described in literature as the second victim phenomenon [14]. This concept pertains to healthcare professionals who, following an adverse clinical event, experience considerable psychological distress, including feelings of shame, self-doubt, and hypervigilance, which subsequently influence their clinical decision-making [15]. Thus, such reactions are not confined to personal suffering but may also affect professional functioning, including confidence in clinical judgment, therapeutic engagement, and tolerance of clinical risk.

Several theories provide explanations for the diverse professional responses observed following a patient’s suicide. One potential pathway is post-traumatic growth, in which clinicians develop heightened reflective capacity, recalibrate professional expectations, and gain enhanced clinical insight as they integrate the experience into their practice [16,17]. Conversely, psychiatrists’ grief is often disenfranchised, as their loss is frequently unrecognised or regarded as less legitimate than that of patients’ families, thereby restricting opportunities for emotional validation and support [17,18]. This phenomenon may contribute to internalised guilt and a reluctance to seek assistance [19]. In conjunction with these processes, clinicians may engage in defensive medical practices, characterised by fear-driven increases in vigilance, excessive documentation, or avoidance of perceived high-risk cases, with the intent of self-protection rather than therapeutic benefit [5,20].

Notably, most of the existing literature concerning the impact of patient suicide on clinicians originates from high-income countries, with limited data available from low- and middle-income countries (LMICS). In Malaysia, classified as an LMIC, the psychiatric workforce remains limited, with approximately 600 psychiatrists serving a population exceeding 33 million. Despite this, there has been minimal research examining the emotional and professional impact of patient suicide among Malaysian psychiatrists, as well as the support structures accessible to them [21]. This survey conducted among psychiatrists in Malaysia was conducted to explore their experiences following patient suicide and to understand their support needs in the aftermath. The findings aim to identify gaps in existing postvention systems and to inform the development of locally relevant clinician-support guidelines.

## 2. Materials and methods

### 2.1 Study design

A cross-sectional survey was conducted among psychiatrists practicing in private, university, and government settings across Malaysia. The study aimed to examine the personal and professional impact of patient suicide on psychiatrists in Malaysia. Data collection began in 1^st^ December 2023 and concluded in 30^th^ April 2024.

### 2.2 Participants and sampling

At the time of the study, Malaysia had approximately 600 practising psychiatrists. Snowball and convenience sampling methods were used for participant recruitment. Psychiatrists were invited through professional networks, institutional mailing lists, and direct referrals from colleagues. The survey invitation was disseminated through a national psychiatric association WhatsApp group comprising approximately 323 members and was also sent to 30 Heads of Psychiatry Departments in Malaysian universities for further distribution. As convenience and snowball sampling methods were employed and the survey link could be redistributed beyond the original recipients, the total number of psychiatrists who received the invitation could not be determined. Therefore, a formal response rate could not be calculated. Eligible qualified participants included psychiatrists currently practicing in private hospitals, university hospitals, and government healthcare facilities in Malaysia. The study did not include psychiatry trainees.

### 2.3 Data collection

Data were collected using a self-administered online questionnaire, which was adapted from the survey used in a previous study examining the impact of patient suicide on clinicians [3]. The questionnaire covered several domains such as sociodemographic information (e.g., age, gender, years of practice, workplace setting), details of the suicide event, personal and professional impact, helpful and unhelpful actions, resources and coping mechanisms. Several modifications were made to improve relevance to the Malaysian context, including changes to demographic and professional characteristics, removal of UK-specific organisational and medico-legal items, and inclusion of culturally relevant support options. A pilot test was conducted to evaluate the survey within the Malaysian context. The evaluation confirmed the instrument’s feasibility, clarity, and cultural relevance, with only minimal modifications required. The questionnaire was hosted on an online survey platform and distributed via email and professional groups. The questionnaire was administered in English.

### 2.4 Ethical considerations

This study was approved by Medical Research and Ethics Committee (MREC), Ministry of Health Malaysia (MOH) (NMRR ID-24–00228-IJD) and the Universiti Kebangsaan Malaysia Research Ethics Committee (FF-2023–288). All procedures were conducted in accordance with the ethical standards according to the principles of the Declaration of Helsinki. Participation in the study was voluntary. Prior to participation, all potential participants were provided with a detailed participant information sheet outlining the purpose of the study, procedures involved, potential risks and benefits, and their rights as participants. Participants were required to indicate informed consent electronically before accessing and completing the questionnaire. Only participants who provided informed consent were able to proceed with the survey. To protect participant privacy and confidentiality, all responses were collected anonymously. No personal data that could identify participants was collected or stored, and no personally identifiable information was obtained.

### 2.5 Data analysis

Quantitative data was analyzed using descriptive statistics using SPSS. Free-text responses were analysed using an inductive thematic grouping approach. Two authors (MHAL and NIAT) independently reviewed the responses and identified preliminary codes and themes. The emerging themes were subsequently discussed and refined through consensus until agreement was reached. The final themes were used to summarise psychiatrists’ experiences of patient suicide, its impact on clinical practice, and perceived support needs.

## 3. Results

### 3.1 Participant characteristics

A total of 120 psychiatrists practicing across Malaysia participated in the study. The median age of respondents was 38 years (IQR 36.8–42.3 years), and the median duration of psychiatric practice was 3 years (IQR 1–10 years). Among the respondents, 44.2% were male, while 55.8% were female. In terms of ethnicity, the majority identified as Malay (60%), followed by Chinese (25%), Indian (12.5%), Punjabi (1.7%), and Native (0.8%). The majority were employed in government hospitals (56.7%), while others worked in academic institutions (22.5%) and private practice (27.5%). The majority of respondents worked in both outpatient and inpatient settings (63.3%), while (25.8%) only worked in outpatient settings.

Seventy-nine percent (n = 95) had experienced at least one patient suicide. Over half (53.7%) reported that the most impactful suicide occurred within their first five years of practice with 17.9% of these happening within the first year, often in community (51.6%) or clinical (48.4%) settings

### 3.2 Experience of psychiatrists following patient suicide

#### 3.2.1 Effects on clinicians’ emotional well-being and mental health.

Respondents were asked to rate the effect of the death on their emotional well-being on a Likert scale, where 0 = ‘not affected at all’ to 10 = ‘very affected by the event’. Most (N = 66, 69.5%) rated their experience above 5, with a median rating of 7 out of 10. The majority (92.6%) felt that their symptoms did not meet a clinical threshold for diagnosis of a psychiatric disorder at any time, one respondent felt he/she met clinical diagnosis, while 6 (6.3%) participants were uncertain. When asked to describe the primary emotional effect of the death, all 95 psychiatrists who experienced this situation reported experiencing painful emotions, such as sadness (N = 59, 62.1%); regret (N = 28, 29.5%); guilt (N = 21, 22.1%); shame (N = 11, 11.6%); anger (N = 7, 7.4%); shock (N = 9, 9.5%); and worried (N = 5, 5.3%). Despite the distress reported, the majority of participants (N = 91, 95.7%) did not take any time off work following the event.

#### 3.2.2 Effects on clinical practice.

Regarding the impact on clinical practice, 56.8% of participants reported a detrimental effect on their confidence. Among those affected, the duration of this impact varied; for 31.6% (N = 30), it lasted between one and four weeks, while 18.9% (N = 18) experienced it for one to six months. A smaller proportion, 2.1% (N = 2), reported the effect persisting for six months to two years, and 4.2% (N = 4) indicated that it was ongoing. Psychiatrists who reported a detrimental effect on their clinical confidence had significantly higher emotional impact scores than those who did not report such effects (p < 0.001). Additionally, more women (N = 34) than men (N = 20) reported a detrimental effect on their clinical confidence following a patient suicide, though the difference was not statistically significant.

Among the 120 participants, 95 (79%) of them who had experienced a patient suicide provided free-text responses. The free-text responses indicate that many psychiatrists experienced a significant impact on their clinical practice. Table 1 summarises the themes describing how psychiatrists’ clinical practice was affected.

**Table 1:** Effect of client suicide on clinical duties.

Themes	Participants’ Responses
Increased vigilance and caution (N = 53/95, 55.8%)	*• “To be more careful with patient presented with almost the same symptoms”* *• “It had taught me to become more vigilant in assessing for potential suicide risk during a patient review”* *• “Documentation becomes more vigorous”*
Reflection and learning from the experience (N = 26/95, 27.4%)	*• “It made me learn and be humble that sometimes things are outside our control. We can try and strive towards zero suicide but just like a myocardial infarct we can do as much the rest is really out of our control”* *• “Spurred me to examine what I could have done or not done to improve overall risk assessment and making better clinical decisions for my future patients.”* *• “Increased regular introspection”*
Increased understanding on suicide risk (N = 18/95, 18.9%)	*• “Understand the importance of suicide risk assessment and support system of the patient”* *• “It made me more aware that suicide risk is rather dynamic, and therefore, it is helpful to revisit it from time to time to ensure we can communicate this clearly to our colleagues who are taking care of the patient and other caregivers.”*
No significant impact (12/95, 12.6%)	*• “Not really affected me too long. The suicide was due to an unexpected social reason”*
Loss of confidence in clinical practice (N = 4/95, 4.2%)	*• “Guilt feeling sometimes roaming around and causing anxiousness when met patient with suicidality”* *• “Impacted self-confidence to practice.”* *• “I lost confidence in managing similar cases in the beginning”*

Career-related impacts following a patient’s suicide were less common among respondents. A small proportion (8.4%, N = 8) considered changing their career due to the experience, while an even smaller group (4.2%, N = 4) contemplated relocating their practice.

#### 3.2.3 Perceived responsibility for the death.

Respondents were asked to rate their feelings of responsibility for the patient’s death, both at the time of the event and in hindsight, using a Likert scale from 0 (“not at all responsible”) to 10 (“felt very responsible”). At the time of the event, 47 respondents (49.5%) rated their sense of responsibility above 5, but this number decreased to 16 respondents (16.8%) in hindsight. Over time, feelings of responsibility diminished, with the median score dropping from 5 (SD = 2.71) to 3 (SD = 2.36). There was a statistically significant difference between male and female respondents in their immediate feelings of responsibility (p = 0.01), with female respondents reporting higher levels of perceived responsibility at the time of the event compared to males. There is a strong positive correlation between psychiatrists’ emotional well-being and perceived responsibility (r = 0.600, N = 95, p < 0.001), suggesting that higher levels of emotional distress are associated with higher levels of perceived responsibility following a suicide incident.

#### 3.2.4 Beliefs and perceptions on suicide prevention.

Psychiatrists rated the extent to which they felt suicide prevention was their role on a Likert scale from 0 (“not agree at all”) to 10 (“very fully agree”). The majority (N = 86, 71.7%) rated their responses above 5, indicating agreement that suicide prevention is their professional responsibility. When asked about the pressure they felt from external sources to prevent patient suicide on a Likert scale from 0 (“not feeling pressure at all”) to 10 (“feeling very pressured”), the median score was 7. A total of 84 respondents (70.0%) scored above 5, indicating that a significant majority of psychiatrists experience moderate to high levels of external pressure in their role. Additionally, psychiatrists were asked to what degree they believed suicide was predictable, using a scale ranging from 0 (“cannot predict at all”) to 10 (“very easily predictable”). The median score for this question was 6, with 64 respondents (53.3%) rating above 5, suggesting that more than half believed suicide was at least somewhat predictable.

#### 3.2.5 Perceptions of the formal processes following the death.

Among those who had lost a patient to suicide, 53.7% were not well-acquainted with the procedures following a suicide event.

### 3.3 Support following patient suicide

About 16.8% of psychiatrists accessed external support outside of work, such as from a spouse or close friends. Those who received external support were associated with a significantly greater impact on their emotional well-being following the loss compared to those who did not (p = 0.011). The free-text responses on helpful and unhelpful support received after the loss are summarized in themes and presented in Table 2.

**Table 2:** Helpful and unhelpful support received after the loss.

Helpful	Unhelpful
Supportive leadership and supervision *• “How the management and bosses managed the treating team. No finger-pointing at all throughout the process. I felt supported.”*	Blaming *• Yeah, being blamed for it. Only recently I was being asked again “what did you say to that patient that caused him to suicide?”*
Peer and collegial support *• “Experience sharing from my other colleagues experiencing similar situations in the past has helped me a lot to process what has happened”*	Lack of organisational support and structure *• “Asked to attend the hospital meeting regarding this case alone by the head of department without any other senior representation from my department”*
Family, friends, and social support *• “Talking about it, support from family and friends, spiritual intervention”*	Unsupportive leadership *• “Superiors who ignore and minimize it”*
Clear procedures and organisational structure *• “We had clear guidelines on what to do after a patient suicide and the resources available”*	Unhelpful self-internal responses *• “My own ruminating thoughts about what could have been done differently to the deceased seemed to be unhelpful. Some things are beyond my control.”*
Personal coping and spirituality *• Faith knowledge and humility* *• Religion*	
Learning and professional growth *• Experience* *• Learning psychiatry* • *Self-learn*	

Respondents were asked about the types of support they would want following a patient suicide, with the option to select multiple responses. The findings are presented in the Table 3.

**Table 3:** Support wanted after a patient suicide.

Support wanted after a patient suicide (N = 120)	
Personal debriefing	81 (67.5%)
Help in communicating or meeting the family/ friends of the patient who has died (e.g., head of department (HOD) or senior colleague)	71 (59.2%)
A senior clinician with a role as suicide lead who could give confidential advice and support	62 (51.2%)
Information about resources for families affected by suicide	62 (51.2%)
Information about the process following patients’ death by suicide	61 (50.8%)
Organised peer support	61 (50.8%)
A confidential reflective practice group or space specifically for processing the effects of a patient suicide	60 (50.0%)
Support for the formal processes following a patients suicide	58 (48.3%)
A training session about this topic	50 (41.6%)
Information about suicide from cultural and spiritual context	42 (35.0%)
Information about support for the community (including schools)	42 (35.0%)
A workshop to share experiences	40 (33.3%)
Counselling and therapy	23 (19.2%)

## 4. Discussion

### 4.1 Experience of psychiatrists following patient suicide

Among psychiatrists who participated in this study, the majority reported having experienced the loss of a patient to suicide. These findings are consistent with studies from the United Kingdom and Thailand [4,22]. Several studies suggest that early-career clinicians are particularly vulnerable to the emotional impact of patient suicide due to clinical inexperience and limited exposure to coping strategies during training [8,23].

Common emotional responses reported in this study included sadness, guilt, and regret, which are consistent with findings from studies conducted in the United Kingdom, Saudi Arabia, and across multiple countries in systematic reviews [3–5,24]. Although these emotional reactions appear relatively consistent across cultural settings, coping strategies, help-seeking behaviours, and perceptions of support may be influenced by local cultural, religious, and healthcare system factors. These reactions align with grief responses observed among other suicide loss survivors, suggesting that psychiatrists experience comparable bereavement reactions despite their professional role [12]. This study reported a detrimental effect in clinical confidence following the loss. The duration of this effect varied among psychiatrists from weeks to years with some psychiatrists reporting ongoing distress at the time of the study. This variability in impact is consistent with the framework proposed by Cerel et al., which categorises individuals exposed to suicide into four groups: suicide-exposed, suicide-affected, and suicide-bereaved (short-term and long-term), depending on the level of attachment and the personal impact of the loss [13]. Psychiatrists in this study appeared across all categories, reflecting the varied ways in which clinicians process and are affected by patient suicide. This study also found that emotional distress was associated with perceived responsibility for the patient’s death indicating that self-blame may be a mechanism intensifying and prolonging distress following patient suicide [25]. As psychiatrists in Malaysia often assume both clinical and therapist roles, the degree of emotional impact may be influenced by the depth of the therapeutic relationship and perceived responsibility toward the patient.

Female psychiatrists in this study reported greater impact and higher perceived responsibility following patient suicide than male psychiatrists which is consistent with previous findings [4]. This suggested gendered patterns in self-blame and emotional processing among clinicians and may warrant targeted support considerations.

In terms of clinical practice, most psychiatrists in this study reported heightened vigilance in suicide risk assessment following a patient’s suicide. Similar findings have been reported internationally. Studies from the United Kingdom, Switzerland, and Saudi Arabia found that clinicians often become more attentive to suicide risk factors, engage in more detailed risk assessments and documentation, adopt more cautious clinical decision-making, and in some cases demonstrate a greater tendency towards defensive practice or hospitalisation of high-risk patients [3,17,24,26]. The consistency of these observations across diverse healthcare systems suggests that increased vigilance may represent a common professional response to patient suicide.

From one perspective, increased vigilance may reflect post-traumatic growth, whereby clinicians adapt following a traumatic professional event through reflective learning and enhanced professional insight [16]. Other themes identified in this study, particularly reflective learning and increased understanding of suicide risk, align with this growth-oriented interpretation. This suggested that clinicians may integrate the experience into their professional identity in ways that strengthen future clinical judgment.

An alternative interpretation is that increased vigilance may reflect hypervigilance, a phenomenon commonly described in post-traumatic stress reactions following highly distressing events. In the context of patient suicide, clinicians may become more alert to suicide risk, engage in repeated checking behaviours, or experience heightened concern about overlooking warning signs. However, as post-traumatic stress symptoms were not directly assessed in this study, it is not possible to determine whether the reported vigilance represents a trauma-related response. Future studies incorporating validated measures of post-traumatic stress may help clarify this relationship.

However, the same behavioural shift may also be interpreted through the perspective of defensive medicine. Defensive clinical practices are typically characterised by increased caution, heightened risk aversion, and extensive documentation, driven primarily by fear of blame, litigation, or professional scrutiny rather than by evidence-based improvements in patient care [20]. In such contexts, increased vigilance may function more as a protective strategy for the clinician than as a mechanism for improving therapeutic outcomes.

Importantly, this study found that increased vigilance often co-occurred with loss of confidence. This pattern mirrors findings from the “second victim” literature, which describes how healthcare professionals involved in adverse events may experience shame, self-doubt, hypervigilance, and emotional distress that influence subsequent clinical decision-making [14,15]. Without adequate postvention support, these reactions may push clinicians toward overly conservative or fear-driven practices that risk undermining therapeutic alliance and clinician wellbeing.

Career-related consequences following a patient’s suicide were relatively uncommon in this study, with only a small minority of psychiatrists who participated in this study considering a change in career or relocation of their practice. Similar consequences have been reported internationally, although at substantially higher rates. In the United Kingdom, 27% of psychiatrists reported considering a change in career following a patient suicide [4], while 35.5% of psychiatrists and psychiatrists in training in Ireland reported similar thoughts [9]. In contrast, only 8.4% of respondents in this study considered changing their career. The low proportion of respondents contemplating career change may also reflect a sense of professional resilience or strong vocational identity within psychiatry. However, it is important to recognise that the absence of career exit does not equate to the absence of harm, as ongoing distress or defensive practice may persist even when clinicians remain in their roles.

The impact of losing a patient to suicide on psychiatrists may be shaped by a combination of personal, professional, and organisational factors. Nearly half of respondents reported moderate to high levels of perceived responsibility immediately following the suicide, although these feelings diminished over time. This pattern could be described as acute self-blame and guilt as early reactions that may gradually lessen as clinicians engage in reflection and cognitive reappraisal. The strong positive association between emotional distress and perceived responsibility further indicates that heightened self-blame may function as a marker of psychological vulnerability rather than objective culpability. This interpretation is consistent with findings from a national survey of French psychiatrists, in which the feeling of responsibility for the death was strongly associated with negative emotional impacts [11], and with qualitative evidence showing that self-blame and self-depreciation in trainees following patient suicide reflect maladaptive psychological responses rather than accurate assessments of fault [27]. This aligns with the broader bereavement literature, in which self-blame has been identified as a powerful determinant of grief-related difficulties [28], and with psychodynamic frameworks describing how guilt and internal persecution in clinicians following patient suicide are driven by psychological defence mechanisms rather than objective culpability [25].

Professional beliefs and systemic pressures also appear to shape the impact of patient suicide. Most psychiatrists viewed suicide prevention as a core professional responsibility and reported substantial external pressure to prevent suicide, alongside beliefs that suicide is at least somewhat predictable. These findings reflect a broader pattern described in the literature, wherein psychiatrists are commonly expected to assess and manage suicide risk as a primary professional duty. It is a role so deeply embedded in professional identity that when a patient dies by suicide, clinicians’ core assumptions about their competence and capacity to heal are often profoundly challenged [29,30]. Indeed, unreasonable systemic expectations of suicide predictability have been shown to directly amplify clinicians’ sense of responsibility following a patient’s death, reinforcing an unrealistic perception of control that is unsupported by evidence on the inherent limitations of suicide risk prediction [31]. These perceptions may amplify distress when a suicide occurs, contributing to moral distress. Moral distress is defined as the psychological unease experienced when a professional perceives themselves as unable to fulfil what they regard as an ethical obligation, despite having acted appropriately [32] or driving defensive clinical practice. The latter has been empirically demonstrated in surveys of psychiatrists, where perceived professional liability for suicide outcomes was directly associated with higher rates of defensive medicine, particularly among younger and less experienced clinicians [33]. Additionally, more than half of psychiatrists were unfamiliar with formal procedures following a patient’s suicide, highlighting organisational gaps that may exacerbate uncertainty and emotional burden.“

### 4.2 Support following patient suicide

Findings from this study highlight substantial gaps between psychiatrists’ support needs following a patient suicide and the support that is actually accessed or available within the Malaysian healthcare system. Currently, the treating psychiatrist will be responsible for conducting postvention support for the family following a suicide. This includes administrative tasks such as mortality review, for which no adequate formal support is provided.

Despite high levels of emotional impact, only a small proportion of psychiatrists sought external support, and the vast majority did not take time off work. This pattern may reflect a culture of professional stoicism, stigma and shame, or fear of being perceived as clinically incompetent, rather than an absence of distress [25].

Workplace-based support emerged as the most salient protective factor. Collegial support and peer discussions were frequently described as helpful, particularly when colleagues had lived experience of patient suicide. Such peer validation may reduce isolation and normalise emotional reactions. The importance of collegial support and supportive leadership identified in this study is consistent with findings from international research. A systematic review by Sandford et al. reported that peer support, opportunities for reflective discussion, and supportive organisational responses were among the most frequently cited protective factors following patient suicide [17]. Similarly, studies from the United Kingdom, Belgium, and Saudi Arabia have found that clinicians value support from colleagues who have experienced similar events, compassionate leadership, and non-blaming workplace cultures [3,24,26,34]. These findings may be understood through the lens of the second victim phenomenon, whereby the psychological impact of an adverse event is influenced not only by the event itself but also by the organisational response that follows.

Supportive leadership and supervision were also critical. Respondents valued senior clinicians and heads of department who adopted non-blaming, transparent, and compassionate approaches. In contrast, blaming attitudes, minimisation of the event, and lack of senior representation during formal reviews were experienced as particularly harmful, intensifying guilt and distress.

Clear organisational procedures following patient suicide were identified as another important form of support. Over half of the psychiatrists reported being unfamiliar with formal processes after a suicide, suggesting systemic inadequacies. Uncertainty around medico-legal procedures, investigations, and communication with families may compound distress and contribute to defensive clinical practices. Access to clear guidelines, defined roles, and structured debriefing processes may therefore serve both emotional and professional protective functions.

Beyond organisational factors, personal coping strategies played a meaningful role. Support from family and friends, personal reflection, and spirituality were commonly cited as helpful. The prominence of spiritual and faith-based coping is particularly relevant in the Malaysian context and mirrors findings from other Asian settings [22]. Similar findings have been reported in other Muslim-majority settings. In Saudi Arabia, nearly 24% of psychiatrists and psychiatry trainees reported using religion or spirituality as a coping strategy following patient suicide or serious suicidal attempts [24], while another study found religion to be among the most commonly utilised coping strategies among psychiatrists [26]. These findings suggest that spiritual and religious beliefs may serve as important sources of meaning-making, acceptance, and resilience following emotionally challenging clinical events. Postvention frameworks developed for Muslim-majority settings may therefore benefit from recognising and incorporating faith-based coping alongside professional and organisational support mechanisms.

Importantly, when asked about desired support, psychiatrists expressed a clear preference for structured, proactive interventions rather than ad hoc or self-initiated help-seeking. Personal debriefing, senior clinician mentorship, organised peer support, confidential reflective spaces, and guidance on communicating with bereaved families were among the most frequently endorsed needs. These preferences suggest psychiatrists are not opposed to support but are limited by a lack of accessible, confidential, and institutionally endorsed postvention services.

Malaysia has an existing national Guideline on Suicide Risk Management in Hospitals, which outlines procedures for suicide prevention, incident reporting, mortality review, and post-incident management following inpatient suicide. The guideline includes recommendations for staff debriefing and counselling after such incidents. However, its primary focus is on patient safety, organisational response, and service improvement within hospital settings rather than on the longer-term emotional impact of patient suicide on psychiatrists or structured clinician-focused postvention support. The findings of the present study highlight the need to strengthen this aspect of postvention care for mental health professionals.

This study has several limitations. First, the cross-sectional design restricts the ability to determine temporal relationships or draw causal inferences. The sample may not be fully representative of the wider psychiatrist population, as recruitment was dependent on approval and access from participating centres, which may have introduced selection bias and limited generalisability. The psychiatrists with particularly strong experiences or views regarding patient suicide may have been more likely to participate. Conversely, those who found the experience highly distressing may have chosen not to participate, potentially influencing the findings. The snowballing method may also lead to a lack of representativeness, as some psychiatrists may not be aware of the survey. Third, the qualitative component was based on free-text responses within the survey rather than in-depth interviews or focus group discussions. As a result, the qualitative data may lack depth and richness, limiting a more nuanced exploration of participants’ experiences and perspectives.

## 5. Conclusion

Patient suicide is a common and profoundly distressing experience for psychiatrists in Malaysia, revealing significant gaps in existing postvention support. Adequate and timely support systems are crucial in enabling psychiatrists to transform potentially traumatic experiences into traumatic growth. The development of structured, targeted (particularly for female and early-career psychiatrists), and proactive local postvention frameworks may support recovery, enhance resilience, and promote professional growth within Malaysian psychiatric practice.
